# Differences in Associated Factors of Underweight and Overweight According to Rural-Urban Residence Strata among Ever-Married Non-Pregnant Women of Reproductive Age in Bangladesh

**DOI:** 10.21315/mjms2024.31.3.18

**Published:** 2024-06-27

**Authors:** Mst Sharmin Akter Sumy, Md Yasin Ali Parh, Most Sifat Muntaha Soni, Nayeem Saifuddin, Jannatul Ferdousi Elma, Hamid Zarei, Md Murad Hossain

**Affiliations:** 1 Department of Bioinformatics and Biostatistics, University of Louisville, Kentucky, USA; 2 Department of Statistics, Islamic University, Kushtia, Bangladesh; 3 Dhaka Nursing College, Dhaka, Bangladesh; 4 Kumudini Nursing College, Mirzapur, Tangail, Bangladesh; 5 Department of Health Management and Systems Science, University of Louisville, Kentucky, USA; 6 Department of Statistics, Bangabandhu Sheikh Mujibur Rahman Science and Technology University, Gopalganj, Bangladesh

**Keywords:** overweight, obesity, underweight, rural women, urban women, Bangladesh

## Abstract

**Background:**

This study sought to compare the prevalence of underweight and overweight among ever-married, non-pregnant women of reproductive age in Bangladesh by urban or rural residency status.

**Methods:**

This study used Bangladesh Demographic and Health Survey (BDHS), 2017 data. Cross-sectional study design with two-stage stratified sampling method was employed. A sample of ever-married non-pregnant women of reproductive age was selected and multinomial logistic regression was utilised in analysis.

**Results:**

It was found that around half of rural women (45.0%, *N* = 4,934) and more than half of urban women (60.3%, *n* = 3,913) were overweight. Nearly one in seven rural women (14.0%, *n* = 1,537) and 1 in 12 urban women (9.0%, *n* = 564) were reported as underweight. Our analyses revealed that being overweight was substantially connected with age, husband’s occupation, economic status, television access, and division for both urban and rural areas. Women from poor households were significantly more likely to be underweight than women from middle- income households for both urban (*P* < 0.05; OR: 1.41; 95% CI: 1.03, 1.94) and rural (*P* < 0.05; OR: 1.23; 95% CI: 1.04, 1.46) areas. Interestingly, women without television access both in urban (*P* < 0.001; OR = 0.78; 95% CI: 0.67, 0.91) and rural (*P* < 0.001; OR = 0.75; 95% CI: 0.68, 0.84) areas had an inverse association with overweight/obesity compared to women with television access. In both areas, women in Sylhet and Mymensingh had higher likelihood of being underweight than Barisal division. Additionally, in both residential zones, women in Sylhet had lower likelihood of being overweight than Barisal division.

**Conclusion:**

This study reveals that multiple characteristics are linked to both overweight and underweight among ever-married, non-pregnant women of reproductive age in Bangladesh. Addressing these variables should be a priority in public health efforts to combat the dual challenge of malnutrition in Bangladesh.

## Introduction

The Sustainable Development Goals (SDGs) aim to eradicate all types of malnutrition by 2030, including undernutrition, overnutrition and non-communicable diseases (NCDs) linked to diet (1). According to the World Health Organization (WHO), communicable diseases, maternal and perinatal disorders, and nutritional deficiencies together accounted for around half of all fatalities in low- and middle-income countries (LMICs) in 2016 (2). Prior to the last 10 years, the focus was on undernutrition in LMICs and on overweight/obesity in high-income countries (3, 4). However, more recently, communities in many LMICs, which have traditionally grappled with undernutrition, have witnessed a significant rise in overnutrition. This phenomenon has given rise to the ‘double burden’ of malnutrition in the majority of LMICs, especially in South Asia (3, 5, 6).

One of the most significant aspects of the demographic shifts that have characterised the 20th century has been the transition in terms of health. Research reveals that the problem of being overweight is progressively surpassing that of being underweight in both rural and urban populations in developing countries as a result of changes in dietary patterns, lifestyle choices and working cultures (7). Thus, public health initiatives in developing countries should address the dual challenges posed by health issues arising from both underweight and overweight individuals (8). According to an estimate by the WHO, 650 million people were underweight and 1.9 billion adults (those aged 18 years old or older) were overweight in 2016 (9). There are both short- and long-term negative effects associated with malnutrition (such as underweight and overweight) (10). For example, being overweight is linked to many gynaecological issues, NCDs and numerous chronic illnesses in women of all ages (11–16).

Despite the global decline in the frequency of underweight people, several LMICs are particularly affected by the trend of rising obesity levels (4, 17–19). An intergenerational cycle of hunger and poverty is known to exist in Bangladesh due to the high prevalence of maternal undernutrition, particularly among teenage girls (17). Moreover, while the nutritional deficits observed among Bangladeshi women have significantly decreased over the past 20 years, chronic undernutrition remains a serious health issue (20). Reports indicate that 22% of women have a vitamin B12 deficiency and 55% of non-pregnant women suffer from zinc deficiency in Bangladesh (21, 22). Studies also show worrying increases in the frequency of overweight among Bangladeshi women of reproductive age (i.e. 15 years old–49 years old). Between 1996 and 2011, there was a substantial increase in the prevalence of overweight among urban women, rising from 11.4% to 28.9%. By contrast, among rural women during the same period, the prevalence of overweight increased from 1.7% to 12.1% (20, 22).

The 2014 Bangladesh Demography and Health Survey (BDHS) provides data concerning the nation on a broad scale, including revealing that 33% of women in rural areas and 53% of women in urban areas are classified as overweight or obese. In addition, about one in eight urban women (12%, *n* = 571) and about one in five rural women (21%, *n* = 2,490) are underweight (23). The BDHS gathers participants’ anthropometric information (i.e. height and weight) through home visits by skilled field research personnel who use data collection techniques that are standardised in the survey setting. A solar-powered electronic scale is used to measure participants’ weight, while an adjustable measuring board is used to determine their height in cm. It is essential to identify personal and household risk factors for underweight or overweight/obese that are influenced by socioeconomic characteristics, including educational attainment, financial position and place of residence (24). The discussion concerning underweight and overweight ever-married, non-pregnant women of reproductive age is well-documented on a global scale, although this issue and its connection with women’s health is yet to be widely explored in Bangladesh.

Most studies investigate the frequency of underweight and overweight/obese among women of reproductive age and elucidate potential socioeconomic variables linked to these conditions (4, 25–27). To develop effective intervention programmes for vulnerable groups, it is crucial to comprehend the differences in the correlations between deterministic factors and the condition of being underweight, overweight or obese in ever-married, non-pregnant women of reproductive age and the situations in this regard in urban and rural areas should be examined separately.

The National Heart, Lung and Blood Institute (NHLBI) states that an individual’s body mass index (BMI) is determined by dividing their weight in kilograms by the square of their height in metres (kg/m^2^) (28). Individuals can be classified according to their BMI based on both Asian-Pacific cutoff points and the conventional WHO classification. The Asian-Pacific BMI cutoff criteria categorise individuals as either underweight (< 18.5 kg/m^2^), normal weight (18.5 kg/m^2^ –< 23.0 kg/m^2^) or overweight/obese (≥ 23.0 kg/m^2^) (29). Individuals can also be categorised into four groups according to their BMI based on the conventional WHO classification: underweight (< 18.5 kg/m^2^), normal weight (18.5 kg/m^2^–24.9 kg/m^2^), overweight (25 kg/m^2^–29.9 kg/m^2^) or obese (≥ 30 kg/m^2^) (30). We used the Asian-Pacific cutoff points to classify participants in our study.

Furthermore, to the best of our knowledge, no prior research has examined the disparities between urban and rural areas in terms of the relationship between sociodemographic characteristics and underweight or overweight/obese among ever-married, non-pregnant Bangladeshi women. To address this gap in the literature, this study used a nationally representative sample of ever-married, non-pregnant women of reproductive age in Bangladesh to determine the differences in the prevalence of underweight and overweight/obese and the associated sociodemographic factors according to the participants’ urban or rural residency status.

## Methods

### Data Sources, Study Design and Sample Size

The data utilised in this study were gathered through the 2017–2018 BDHS, which was carried out by the National Institute of Population Research and Training (NIPORT) under the guidance of the Ministry of Health’s Medical Education and Family Welfare Division of Bangladesh. The survey’s sampling frame was determined from the list of enumeration areas (EAs) included in the 2011 National Population and Housing Census (NPHC). It employed a two-stage stratified sampling strategy, with the first stage consisting of 675 EAs, 250 of which were in urban areas and 425 in rural regions. The second sampling stage involved the deliberate selection of 30 households from each EA. One in three of the 20,250 households that were surveyed were randomly selected for anthropometric testing. The 2017–2018 BDHS report provides more information on the specifics of the survey design, sampling technique, sample size calculation, questionnaire, data collection procedures, and outcomes (12). We included all ever-married, non-pregnant women of reproductive age in our analysis. More details concerning the sample are presented in Table 1.

### Dependent Variable

The dependent variable is ordered as normal, underweight, overweight/obesity which is based on the BMI.

### Explanatory Variables

This study considered demographic, socioeconomic and geographic factors while examining the BMI of ever-married, non-pregnant women of reproductive age who were stratified by residency in urban or rural areas. Based on the data structure, published research and biological plausibility, the following explanatory variables were considered: age at the time of the survey (15–19, 20–29, 30–39 or 40–49) years old, educational status (no education, primary, secondary or higher), husband’s occupation (carpenter, businessman, doctor, farmer, other or unemployed), respondent’s occupation (business/work, carpenter, non-agricultural work, other or unemployed), use of contraceptives (traditional, modern or no method), wealth index (poor, middle or rich), television access (access or no access) and residence by division (Barisal, Chittagong, Dhaka, Khulna, Mymensingh, Rajshahi, Rangpur or Sylhet).

### Statistical Analysis

A descriptive analysis was first performed to determine the sample characteristics according to the respondents’ BMI category and place of residence (urban or rural). Next, the 95% confidence interval (95% CI) and the prevalence of both underweight and overweight were calculated. In addition, multinomial logistic regression analyses were conducted to obtain the odds ratios (ORs) and 95% CIs for the factors of interest. Statistical Package for the Social Sciences (SPSS) version 25.0 and R version 4.0.0 were used for the purpose of data management and analysis.

## Results

### Characteristics of the Study Sample

The respondents’ demographic details, as stratified by BMI category and urban versus rural residency, are shown in Table 1. The analysis was performed on a weighted sample of 17,564 ever-married, non-pregnant women of reproductive age (15 years old–49 years old), 6,484 of whom were from urban areas and 11,080 from rural areas. The majority of underweight women from both rural and urban areas were in the second age group (20 years old–29 years old), whereas the majority of overweight or obese women from both areas were in the third age group (30 years old–39 years old). In both rural and urban areas, the majority of respondents’ husbands earned their living as businessmen or carpenters or through ‘other’ work. Metropolitan areas had a somewhat larger proportion of women who utilised contraception than rural areas. Moreover, in rural areas, more than half of the underweight women were from the poor quintile (59.2%, *n* = 910), whereas nearly half of the overweight or obese women in urban areas were from the rich quintile (48.9%, *n* = 1,757). While the majority of underweight respondents from urban areas were from Dhaka division, the majority of underweight women from rural areas were from Sylhet and Mymensingh divisions.

**Figure 1:**
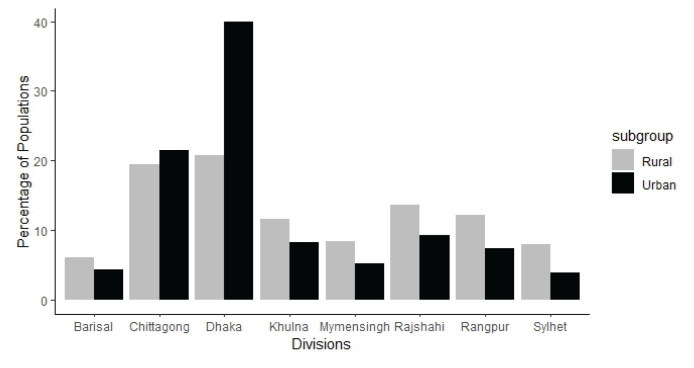
Percentage of populations of different divisions stratified by urban and rural areas in Bangladesh

The distribution of the sample by the location (rural or urban) of the different divisions is shown in Figure 1. Among the divisions, the highest rural population was found in Dhaka (20.74%) and the lowest in Barisal (6.02%). Similarly, the highest urban population was recorded in Dhaka (39.88%), although the lowest was in Sylhet (3.97%).

**Figure 2:**
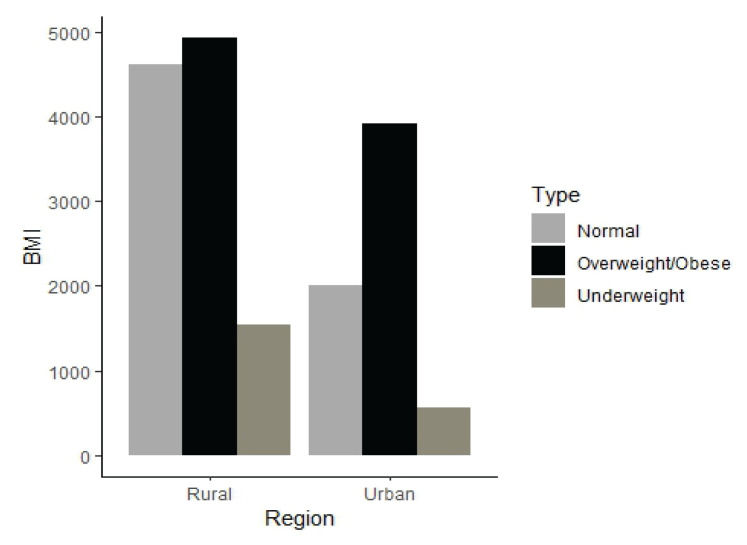
BMI categories of ever-married non-pregnant women of Bangladesh stratified by urban and rural regions

Furthermore, Figure 2 presents the BMI categories by the respondents’ place of residence. When comparing the BMI categories in both areas, it can be seen that the ever-married, non-pregnant women of reproductive age from rural areas had a higher risk of being both underweight and overweight than the women from urban areas.

### Determinants of Overweight

Overall, our analyses revealed that there were differences between urban and rural women as well as different risk factors associated with being overweight (Table 2). Our research found a substantial positive relation between overweight and both increasing age and the wealth index among ever-married, non-pregnant women of reproductive age from both areas. Although both rural and urban residents were more likely to be overweight as they aged, the association was stronger among the urban residents. In urban areas, when compared with women aged 15 years old–19 years old, the odds of being overweight were increased about two-fold among women aged 20 years old–29 years old (OR = 2.78; 95% CI: 2.11, 3.65) and 30 years old–39 years old (OR = 4.86; 95% CI: 3.67, 6.44) years and about five-fold among women aged 40 years old–49 years old (OR = 5.70; 95% CI: 4.22, 7.72]) years. Women whose husbands were doctors were more likely (OR = 1.43; 95% CI: 1.09, 1.89) to be overweight than women whose husbands were businessmen. Women who were married to farmers (OR = 0.75; 95% CI: 0.65, 0.87) were found to have an inverse association with being overweight/obese when compared with women who were married to businessmen, albeit only in rural areas. Women from the wealthy class had a higher chance of being overweight than women from the middle class in both urban (OR = 1.92; 95% CI: 1.59, 2.32) and rural (OR = 1.35; 95% CI: 1.19, 1.53) areas. Interestingly, women without access to television in both urban (OR = 0.78; 95% CI: 0.67, 0.91) and rural (OR = 0.75; 95% CI: 0.68, 0.84) areas had an inverse association with being overweight/obese when compared with women who had access to television. In both urban and rural areas, women from Sylhet (urban: OR = 0.54; 95% CI: 0.40, 0.71); rural: OR = 0.60; 95% CI: 0.50, 0.74) had a lower likelihood of being overweight than women from Barisal division.

### Determinants of Underweight

Age, wealth index and division all had a significant association with underweight among women from both urban and rural areas. Indeed, in both urban and rural areas, women belonging to the poor wealth index group had an increased likelihood of being underweight (urban: OR = 1.41; 95% CI: 1.03, 1.94); rural: OR = 1.23; 95% CI: 1.04, 1.46) when compared with their middle-class counterparts. In both residential areas, women were more likely to be underweight in Mymensingh and Sylhet divisions than in Barisal division. In both urban and rural areas, women from Sylhet (urban: OR = 2.07; 95% CI: 1.29, 3.32; rural: OR = 1.81; 95% CI: 1.41, 2.33) had around a two-fold higher likelihood of being underweight than women from Barisal division. Additionally, in both residential areas, women from Mymensingh (urban: OR = 1.75; 95% CI: 1.07, 2.89; rural: OR = 1.45; 95% CI: 1.13, 1.85) had a higher likelihood of being underweight than women from Barisal.

## Discussion

In the present study, we compared the risk factors for being underweight and overweight/obese among Bangladeshi ever-married, non-pregnant women of reproductive age living in rural and urban areas. The findings revealed that approximately half of the rural women and more than half of the urban women were overweight. By contrast, nearly one in seven rural women and almost one in twelve urban women were found to be underweight. The growing burden of malnutrition in the form of overnutrition among young women in Bangladesh has serious repercussions during both pregnancy and childbirth (31, 32). According to our findings, Bangladeshi ever-married, non-pregnant women of reproductive age are more likely to be overweight than underweight, which is consistent with the findings of previous works. The findings of this study concerning the prevalence of overweight support the hypothesis that, in Bangladesh, the prevalence of overnutrition may surpass that of undernutrition by 2025 (18, 33). Moreover, the findings of this study are consistent with the national estimate of the prevalence of overweight being 39%, which is significantly higher than the prevalence of a prior study based on 2014 BDHS data. Studies have shown that Bangladeshi women are more likely to be physically inactive than women from other South Asian nations (34). Our research indicated a substantial positive correlation between being overweight and of an increasing age among ever-married, non-pregnant women from both area of residence. This finding accords with research conducted in other Asian nations, such as India, which suggests that older women are less likely to engage in physical activity and more likely to consume an obesogenic diet (35).

Most Bangladeshi women are dependent on their husband’s income and are more likely to adopt sedentary lifestyles and to consume more foods that are high in energy but low in nutrients, both of which raise the likelihood of being overweight (36). This pattern of food consumption and lifestyle is common among urban women, which could be the reason for the higher likelihood of being overweight seen among the women from urban areas whose husbands are doctors. Previous studies have observed a positive association between television watching and being overweight. Indeed, television watching is *predictive of both obesity and weight gain because of the associated* sedentary behaviours *and inadequate physical activity levels*, which have been documented as risk factors for overweight. Unlike other forms of mass communication, watching television is regarded as a luxury activity that is typically engaged in while seated. This sedentary behaviour is also associated with the consumption of unhealthy food, particularly high-calorie fast food (37). By contrast, women who do not watch television or read newspapers or magazines have a higher probability of being underweight (37), which was also found in our study. In this study, we further found that rural women who use traditional contraceptives and who do not use any contraceptives are at risk of being underweight. Those who had never used contraceptives were more likely to report being underweight, which is consistent with previous reports (38). However, we only observed this association for rural women, with the probable cause being that rural women are less educated and not as conscious of their health as urban women.

### Strengths and Limitations

It must be acknowledged that this study has both strengths and limitations. By using the Asian-Pacific BMI cutoffs and stratifying the findings by rural–urban location, this is the first nationally representative epidemiological study of Bangladeshi ever-married, non-pregnant women of reproductive age to investigate the prevalence and determinants of respondents’ body weight status. The assumption of causal relationships between the explanatory determinants and outcomes is somewhat restricted due to the cross-sectional nature of the data. Moreover, the sample was divided into categories using the Asian-Pacific BMI cutoffs, which, when compared with the BMI cutoffs recommended by the WHO, can exaggerate the overweight population.

## Conclusion

This study highlights the significant prevalence of the ‘double burden’ associated with overweight and underweight status among ever-married, non-pregnant women of reproductive age in both urban and rural areas of Bangladesh by utilising nationally representative data. Our results showed that, for both urban and rural areas, being overweight was significantly correlated with the respondents’ age, husband’s occupation, economic status, access to television, and division. When compared with women from rural areas, women from urban areas were more likely to be overweight. In both urban and rural areas, women from low-income households were substantially more likely to be underweight than women from middle-income homes. Furthermore, women who did not have access to television in both urban and rural areas had a negative association with being overweight/obese when compared with those who did have access to television. A thorough public awareness campaign and both practical and cost-effective health interventions must be developed as part of Bangladesh’s national health policy to address the growing number of people with a non-normal weight status. Additionally, the significant incidence of underweight young women from both rural and urban areas necessitates the prioritisation of specific health interventions for this population.

## Figures and Tables

**Table 1:** Sample characteristics according to body weight in urban and rural regions

	Urban (*N* = 6,484)			Rural (*N* = 11,080)		

	Normal (*n* = 2,007)	Overweight (*n* = 3,913)	Underweight (n = 564)	Normal (*n* = 4,609)	Overweight (*n* = 4,934)	Underweight (*n* = 1,537)
	*n* (%)	*n* (%)	*n* (%)	*n* (%)	*n* (%)	*n* (%)
Age (years old)						
15–19	211 (10.5)	121 (3.1)	106 (18.8)	500 (10.8)	182 (3.7)	223 (14.5)
20–29	772 (38.5)	1208 (30.9)	227 (40.2)	1533 (33.3)	1425 (28.9)	521 (33.9)
30–39	604 (30.1)	1485 (38.0)	121 (21.5)	1457 (31.6)	1924 (39.0)	410 (26.7)
40–49	420 (20.9)	1099 (28.1)	110 (19.5)	1119 (24.3)	1403 (28.4)	383 (24.9)
Respondent’s education						
No education	347 (17.3)	456 (11.7)	114 (20.2)	987 (21.4)	766 (15.5)	380 (24.7)
Primary	640 (31.9)	1007 (25.7)	200 (35.5)	1602 (34.8)	1692 (34.3)	569 (37.0)
Secondary	730 (36.4)	1510 (38.6)	187 (33.2)	1657 (36.0)	1965 (39.8)	503 (32.7)
Higher	290 (14.4)	940 (24.0)	63 (11.2)	363 (7.9)	511 (10.4)	85 (5.5)
Husband’s education						
No education	391 (21.3)	531 (14.5)	114 (22.4)	1178 (27.4)	1014 (21.7)	430 (30.6)
Primary	356 (19.4)	509 (13.9)	109 (21.4)	1011 (23.5)	937 (20.0)	341 (24.2)
Secondary	231 (12.6)	371 (10.1)	72 (14.1)	554 (12.9)	554 (11.8)	201 (14.3)
Higher	860 (46.8)	2257 (61.5)	215 (42.2)	1556 (36.2)	2176 (46.5)	435 (30.9)
Respondent’s occupation						
Business/work	98 (4.9)	290 (7.4)	19 (3.4)	105 (2.3)	143 (2.9)	26 (1.7)
Carpenter	147 (7.3)	324 (8.3)	24 (4.3)	185 (4.0)	226 (4.6)	44 (2.9)
Non-agricultural work	322 (16.0)	379 (9.7)	101 (17.9)	158 (3.4)	118 (2.4)	63 (4.1)
Others	458 (22.8)	620 (15.8)	131 (23.2)	2314 (50.2)	2221 (45.0)	772 (50.2)
Unemployed	982 (48.9)	2300 (58.8)	289 (51.2)	1847 (40.1)	2226 (45.1)	632 (41.1)
Husband’s occupation						
Businessman	446 (24.3)	1109 (30.2)	90 (17.6)	703 (16.4)	960 (20.5)	184 (13.1)
Carpenter	499 (27.1)	1002 (27.3)	136 (26.7)	836 (19.4)	985 (21.0)	284 (20.2)
Doctor	122 (6.6)	484 (13.2)	19 (3.7)	95 (2.2)	232 (5.0)	28 (2.0)
Farmer	106 (5.8)	138 (3.8)	36 (7.1)	950 (22.1)	907 (19.4)	276 (19.6)
Others	624 (33.9)	841 (22.9)	212 (41.6)	1628 (37.9)	1490 (31.8)	605 (43.0)
Unemployed	41 (2.2)	94 (2.6)	17 (3.3)	87 (2.0)	107 (2.3)	30 (2.1)
Contraceptive use						
Modern method	1115 (55.6)	2142 (54.7)	301 (53.4)	2464 (53.5)	2561 (51.9)	702 (45.7)
Traditional Method	193 (9.6)	473 (12.1)	58 (10.3)	410 (8.9)	519 (10.5)	164 (10.7)
No Method	699 (34.8)	1298 (33.2)	205 (36.3)	1735 (37.6)	1854 (37.6)	671 (43.7)
Wealth index						
Poor	997 (55.5)	1198 (33.4)	334 (66.5)	2151 (46.7)	1464 (29.7)	910 (59.2)
Middle	370 (20.6)	636 (17.7)	82 (16.3)	952 (20.7)	969 (19.6)	299 (19.5)
Rich	430 (23.9)	1757 (48.9)	86 (17.1)	1506 (32.7)	2501 (50.7)	328 (21.3)
Television access						
No access	778 (38.8)	884 (22.6)	283 (50.2)	3048 (66.1)	2493 (50.5)	1165 (75.8)
Division						
Barisal	198 (9.9)	365 (9.3)	43 (7.6)	509 (11.0)	597 (12.1)	157 (10.2)
Chittagong	267 (13.3)	578 (14.8)	64 (11.3)	577 (12.5)	856 (17.3)	113 (7.4)
Dhaka	461 (23.0)	1009 (25.8)	118 (20.9)	404 (8.8)	531 (10.8)	115 (7.5)
Khulna	254 (12.7)	538 (13.7)	70 (12.4)	570 (12.4)	759 (15.4)	160 (10.4)
Mymensingh	158 (7.9)	259 (6.6)	51 (9.0)	635 (13.8)	487 (9.9)	269 (17.5)
Rajshahi	243 (12.1)	471 (12.0)	58 (10.3)	632 (13.7)	669 (13.6)	211 (13.7)
Rangpur	193 (9.6)	388 (9.9)	58 (10.3)	735 (15.9)	590 (12.0)	237 (15.4)
Sylhet	233 (11.6)	305 (7.8)	102 (18.1)	547 (11.9)	445 (9.0)	275 (17.9)

**Table 2:** Multinomial logistic regression for estimating the OR and 95% CI for being underweight and overweight

	Overweight versus Normal weight OR (95% CI)		Underweight versus Normal weight OR (95% CI)	

	Urban	Rural	Urban	Rural
Age (years old)				
15–19	Ref.	Ref.	Ref.	Ref.
20–29	2.78***(2.11, 3.65)	2.92***(2.41, 3.54)	0.55**(0.40, 0.75)	0.72**(0.59–0.88)
30–39	4.86***(3.67, 6.44)	4.77***(3.91, 5.81)	0.33***(0.23, 0.47)	0.55***(0.45–0.69)
40–49	5.70***(4.22, 7.72)	5.14***(4.15, 6.36)	0.36***(0.24, 0.55)	0.62***(0.49–0.80)
Respondent’s education				
Higher	Ref.	Ref.	Ref.	Ref.
Secondary	1.02 (0.83, 1.25)	1.10 (0.93, 1.32)	0.86 (0.59, 1.26)	1.05 (0.79, 1.39)
Primary	0.90 (0.71, 1.15)	1.05 (0.87, 1.28)	1.06 (0.70, 1.62)	1.14 (0.84, 1.54)
No education	0.75 (0.56, 1.01)	0.76* (0.61, 0.95)	1.17 (0.71, 1.94)	1.19 (0.85, 1.67)
Husband’s education				
Higher	Ref.	Ref.	Ref.	Ref.
Secondary	0.97 (0.78, 1.20)	0.89 (0.77, 1.02)	1.03 (0.72, 1.46)	1.08 (0.88, 1.33)
Primary	0.89 (0.73, 1.08)	0.86*(0.75, 0.97)	1.03 (0.75, 1.41)	0.97 (0.81, 1.16)
No education	0.84 (0.68, 1.03)	0.85*(0.74, 0.97)	0.88 (0.62, 1.24)	0.99 (0.82, 1.20)
Respondent’s occupation				
Business/work	Ref.	Ref.	Ref.	Ref.
Carpenter	1.09 (0.77, 1.55)	1.15 (0.80, 1.64)	0.84 (0.40, 1.77)	0.75 (0.42, 1.36)
Non-agricultural work	0.83 (0.59, 1.17)	0.95 (0.64, 1.43)	1.32 (0.68, 2.56)	1.06 (0.59, 1.91)
Others	0.92 (0.67, 1.25)	1.07 (0.80, 1.45)	1.02 (0.54, 1.93)	1.07 (0.66, 1.73)
Unemployed	1.15 (0.86, 1.52)	1.30 (0.96, 1.75)	1.25 (0.69, 2.29)	1.05 (0.65, 1.69)
Husband’s occupation				
Businessman	Ref.	Ref.	Ref.	Ref.
Carpenter	0.94 (0.79, 1.11)	0.96 (0.84, 1.11)	1.25 (0.91, 1.72)	1.19 (0.96, 1.48)
Doctor	1.06 (0.81, 1.39)	1.43*(1.09, 1.89)	0.73 (0.40, 1.36)	1.46 (0.91, 2.35)
Farmer	0.76 (0.56, 1.04)	0.75***(0.65, 0.87)	1.53 (0.92, 2.53)	1.01 (0.81, 1.25)
Others	0.82*(0.68, 0.98)	0.87*(0.76, 0.99)	1.48*(1.08, 2.02)	1.17 (0.96, 1.43)
Unemployed	0.83 (0.55, 1.27)	0.80 (0.85, 1.10)	2.14*(1.10, 4.16)	1.18 (0.75, 1.87)
Contraceptive use				
Modern method	Ref.	Ref.	Ref.	Ref.
Traditional method	0.99 (0.81, 1.22)	1.07 (0.84, 1.71)	1.19 (0.03, 1.35)	1.50***(1.21, 1.83)
No method	0.91 (0.79, 1.05)	0.99 (0.83, 1.35)	1.06 (0.03, 1.17)	1.37***(1.19, 1.56)
Wealth index				
Middle	Ref.	Ref.	Ref.	Ref.
Poor	0.85 (0.71, 1.01)	0.86*(0.75, 0.97)	1.41*(1.03, 1.94)	1.23*(1.04, 1.46)
Rich	1.92***(1.59, 2.32)	1.35***(1.19, 1.53)	1.12 (0.78, 1.63)	0.68***(0.56, 0.83)
Television access				
	Ref.	Ref.	Ref.	Ref.
No access	0.78***(0.67, 0.91)	0.75***(0.68, 0.84)	1.20 (0.94, 1.53)	1.14 (0.97, 1.35)
Division				
Barisal	Ref.	Ref.	Ref.	Ref.
Chittagong	1.05 (0.81, 1.36)	1.06 (0.89, 1.26)	1.19 (0.74, 1.93)	0.72*(0.54, 0.96)
Dhaka	0.99 (0.77, 1.27)	1.00 (0.83, 1.21)	1.42 (0.89, 2.25)	1.15 (0.86, 1.54)
Khulna	1.01 (0.78, 1.32)	1.04 (0.87, 1.24)	1.62 (1.01, 2.61)	1.13 (0.87, 1.48)
Mymensingh	0.82 (0.61, 1.09)	0.67***(0.57, 0.82)	1.75*(1.07, 2.89)	1.45*(1.13, 1.85)
Rajshahi	1.02 (0.78, 1.32)	0.86 (0.72, 1.02)	1.41 (0.87, 2.30)	1.25 (0.97, 1.61)
Rangpur	1.04 (0.79, 1.37)	0.80*(0.67, 0.95)	1.59 (0.97, 2.58)	1.10 (0.85, 1.40)
Sylhet	0.54***(0.40, 0.71)	0.60***(0.50, 0.74)	2.07**(1.29, 3.32)	1.81***(1.41, 2.33)

Notes: Normal weight was the reference group;

*** *P* < 0.001;

** *P* < 0.01;

* *P* < 0.05

## Data Availability

In this study, we used data from Demographic Health Survey (DHS), which is available from https://dhsprogram.com/data/available-datasets.cfm
